# Ommochrome pathway genes *kynurenine 3-hydroxylase* and *cardinal* participate in eye pigmentation in *Plutella xylostella*

**DOI:** 10.1186/s12860-020-00308-8

**Published:** 2020-09-11

**Authors:** Xuejiao Xu, Tim Harvey-Samuel, Jie Yang, Luke Alphey, Minsheng You

**Affiliations:** 1 State Key Laboratory of Ecological Pest Control for Fujian and Taiwan Crops, Institute of Applied Ecology, Fujian Agriculture and Forestry University, Fuzhou, 350002 China; 2 Joint International Research Laboratory of Ecological Pest Control, Ministry of Education, Fujian Agriculture and Forestry University, Fuzhou, 350002 China; 3 Key Laboratory of Integrated Pest Management for Fujian-Taiwan Crops, Ministry of Agriculture, Fuzhou, 350002 China; 4 Arthropod Genetics Group, The Pirbright Institute, Woking, Pirbright, GU24 0NF UK

**Keywords:** CRISPR/Cas9, *Kynurenine 3-hydroxylase*, *Cardinal*, *Plutella xylostella*, Eye pigmentation, Gene drive

## Abstract

**Background:**

Eye pigmentation genes have been utilized as visible markers for constructing genetic control prototypes in several insect vectors of human disease. Here, orthologs of two ommochrome pathway genes, *kynurenine 3-hydroxylase* (*kmo*) and *cardinal*, were investigated in *Plutella xylostella*, a globally distributed, economically important pest of *Brassica* crops.

**Results:**

Both somatic mosaic and germline mutations were efficiently created using the CRISPR/Cas9 system, and null mutant strains of *Pxkmo* and *Pxcardinal* were obtained. A frame-shift mutation in *Pxkmo* caused yellow compound eyes at adult stage while an in-frame mutation lacking two amino acids resulted in a hypomorphic red eye phenotypes. In contrast, *Pxcardinal*-deficient moths with a frame-shift mutation exhibited yellow eye pigmentation in newly emerged adults which turned to red as the adults aged. Additionally, differences were observed in the coloration of larval ocelli, brains and testes in *Pxkmo* and *Pxcardinal* yellow-eye mutant lines.

**Conclusions:**

Our work identifies the important roles of *Pxkmo* and *Pxcardinal* in *P. xylostella* eye pigmentation and provides tools for future genetic manipulation of this important crop pest.

## Background

The diamondback moth (DBM), *Plutella xylostella*, is one of the most destructive agricultural pests worldwide and causes great economic damage by feeding on cruciferous crops during its larval stage, significantly impacting plant quality and yield [1, 2]. Additionally, its rapid development of resistance against a broad range of insecticides including *Bacillus thuringiensis* (Bt) toxins has become the primary challenge in the management of this global pest [1, 3]. Genetics-based strategies have been proposed as environmentally friendly complements or alternatives to current pesticide-based method. For example, the female-specific RIDL system, where lethal phenotypes are only expressed in females, has been engineered in DBM for reducing female density and consequently suppressing populations [4, 5]. Recently, CRISPR-based gene drive systems have been described in several disease vectors as potentially powerful population suppression/replacement tools, which could be extended to agricultural pest management [6–9]. To construct “proof of principle” models for these novel genetic control systems, phenotypic genes, such as those manipulating body pigmentation or eye coloration, are commonly utilized as primary targets for assessing and improving genome editing capacity. Considering its short life-cycle, ease of rearing and relatively well-annotated genome [10], DBM displays potential as a model agricultural pest for testing genetic-based pest management strategies, including gene drives.

**Fig. 1:**
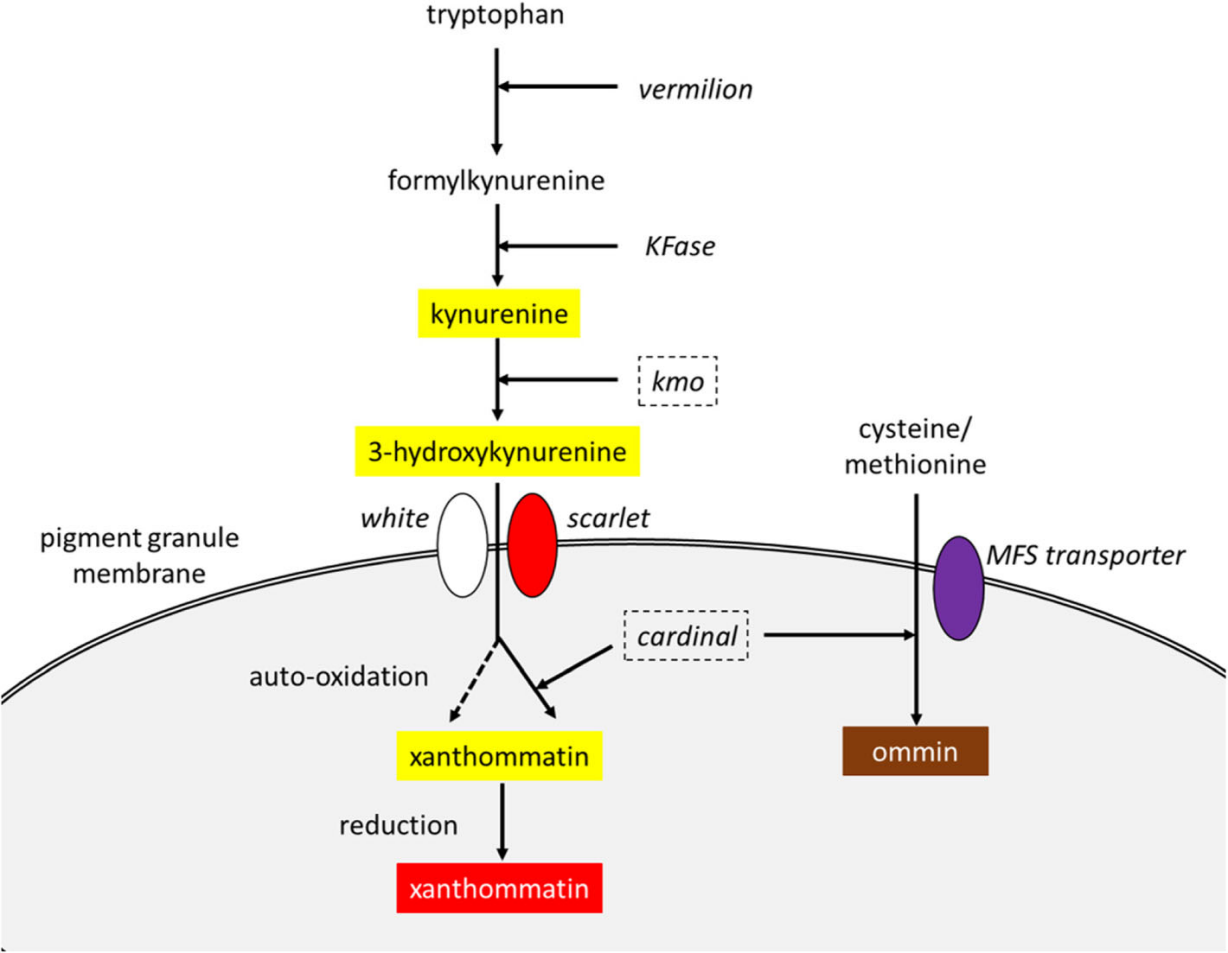
Schematic diagram of ommochrome synthesis pathway in silkworm *B. mori* (edited from [15]). Genes involved in ommochrome pathway are represented in italics, while the *kmo* and *cardinal* genes investigated in this study are indicated with dashed boxes. The color of the pigments/precursors was highlighted with yellow, red or brown boxes

Eye pigmentation genes have attracted great attention for genetic manipulation of insects due to the ease of discriminating viable mutant phenotypes, and in the past, the ease with which these mutants could be rescued by mini-gene constructs [11]. Insect eye color traits are regulated by complex gene networks which vary by species. For example in *Drosophila*, these networks include the ommochrome and pteridine synthesis pathways, while ommochromes are the only visual pigments in other important species such as various mosquitoes, beetles and the silkworm *Bombyx mori* [12–14]. In the *B. mori* ommochrome synthesis pathway, the upstream component tryptophan is oxidized to formylkynurenine by tryptophan oxidase (encoded by *vermilion* in *Drosophila*), after which formylkynurenine is catalyzed into kynurenine by kynurenine formamidase (KFase). Kynurenine is subsequently converted into 3-hydroxykynurenine by kynurenine 3-hydroxylase (kmo, encoded by *cinnabar* in *Drosophila*). The 3-hydroxykynurenine is incorporated into pigment granules by a heterodimer composed of ABC transporters Scarlet and White and then catalyzed into xanthommatin by Cardinal, which is also involved in ommin synthesis (Fig. 1) [14, 15].

In previous reports, different eye-color mutants were generally identified in the field or isolated from laboratory rearing colonies and subsequently identified as possessing mutations in ommochrome pathway genes. For instance, classical mutant strains such as *vermilion*, *cinnabar*, *scarlet*, *cardinal* and *white* were described by early researchers in *Drosophila* [16]. Similarly, *B. mori* white-eyed mutant lines *w-1*, *w-2* and *w-3* were respectively found to correspond to impaired functions of *kmo* (*cinnabar*), *scarlet* and *white* [17–19]. Positional cloning of the *B. mori* egg and eye color mutant *pink-eyed white egg* (*pe*) showed that it was derived from a missense *cardinal* mutation [13]. Spontaneous eye-color mutants have also been reported in *Tribolium castaneum* [20]. These mutant lines provided useful material for studying the metabolism of various ommochrome pigments in insect eggs and eyes. The advance of molecular tools such as RNA interference-mediated gene silencing and nuclease-induced gene disruption has allowed modern researchers to more easily identify these eye-color genes and characterize their biological functions in a range of different insect species [13, 21]. Among these tools, the clustered regularly interspaced short palindromic repeats (CRISPR) / CRISPR-associated protein 9 (CRISPR/Cas9) system has attracted a great deal of attention due to its convenience of design and operation, lower costs, and functionality across different insect species [22].

Despite the essential roles of ommochrome pathway genes *kmo* and *cardinal* in eye pigmentation of some non-drosophilid species [13, 19, 23, 24], the biological functions of these two genes remain unexplored in DBM. In the current study, the CRSPR/Cas9 system, which has been previously demonstrated in DBM [25], was utilized to generate *Pxkmo* and *Pxcardinal* mutant moths. Easily distinguished yellow or red eye colorations were observed in null-mutant adults compared with black compound eyes in wildtype (non-mutated) individuals. Interestingly, in the case of *Pxkmo*, we observed differing color-change phenotypes depending on the nature of the disruption induced (sense or missense mutation). In addition, disruption of Pxcardinal led to an unusual phenotype in adult moths in which eye color gradually changed over time. Our work expands the genome editing toolbox in DBM, providing visible markers for developing ‘proof of principle’ models of novel genetic control tools targeting this global pest.

## Results

### Identification of *P. xylostella kmo* and *cardinal* homologs

We first utilized the annotated *B. mori* kmo protein sequence as a query to blast the DBM genome database, and only one hit (g24581) was found. With reciprocal BlastP of this hit against the *B. mori* genome, only the kmo protein showed high similarity (lowest E value). Using the *D. melanogaster* cardinal protein sequence as a query, several hits containing a conserved peroxidase domain were found in DBM genome, among which g18000 showed the lowest E value. The top four hits for *Drosophila* cardinal in DBM (g18000, g36589, g12936 and g29396) were reciprocally blasted against the *D. melanogaster* genome, and only g18000 gave *Drosophila* cardinal as the most similar hit. Combined with the functional validation below, g24581 and g18000 genes are hereafter named *Pxkmo* and *Pxcardinal*.

**Fig. 2:**
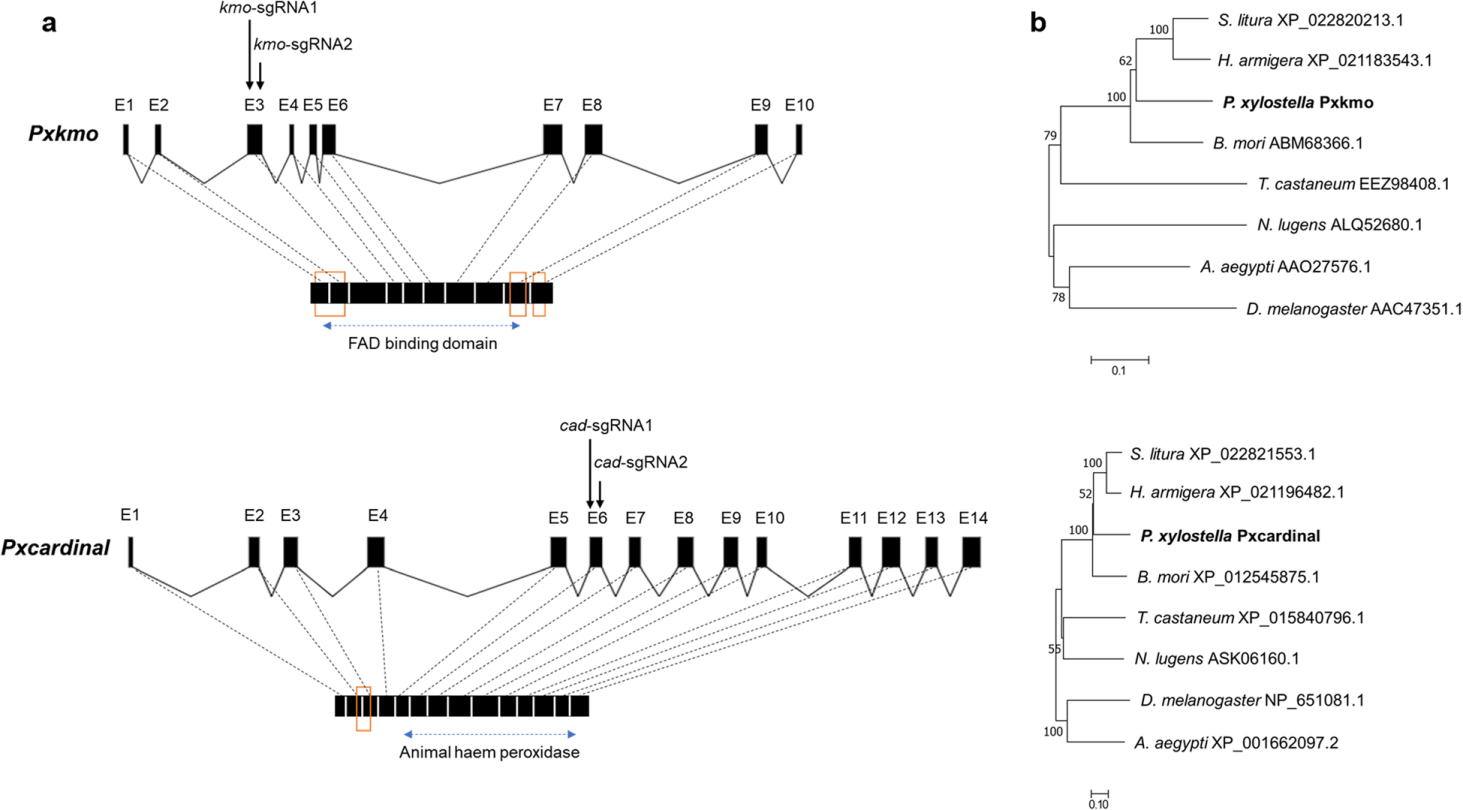
Gene structure (**a**) and phylogenetic analysis (**b**) of *Pxkmo* and *Pxcardinal* **a** Gene structure of *Pxkmo* (upper) and *Pxcardinal* (lower). Predicted splicing patterns are indicated with black boxes (exons) and broken lines (introns). Red boxes and blue dashed lines are used to mark the relative positions of transmembrane structures and conserved domains in translated proteins, respectively. sgRNAs binding sites are indicated with black arrows. **b** Phylogenetic trees of kmo (upper) and cardinal (lower) proteins across insect species (with Maximum Likelihood Method, 1000 bootstraps). Species name (left) and accession number (right) are listed at the terminal of tree branches. Tree scales are provided to indicate the branch length

The coding sequences of *Pxkmo* and *Pxcardinal* were 1344 bp and 2568 bp in length, containing 10 exons and 14 exons respectively. Based on sequence analysis, a FAD binding domain (22–1136 bp) and three transmembrane domains (34–87, 1107–1157 and 1215–1277 bp) were detected in *Pxkmo*, while a peroxidase family conserved domain (742–2397 bp) and one transmembrane domain (163–237 bp) were found in *Pxcardinal* (Fig. 2a).

Phylogenetic trees of *kmo* and *cardinal* homologues were constructed using protein sequences from several lepidopteran, coleopteran, hemipteran and dipteran species. Both Pxkmo and Pxcardinal proteins were clustered with their lepidopteran counterparts (Fig. 2b), consistent with the evolutionary relationships between DBM and the other species compared here [10].

### Cleavage assessment of sgRNA/Cas9 complex

Based on previous reports in other species and analysis with online tool CHOPCHOP, *kmo*-sgRNA1 and *kmo*-sgRNA2 were designed for CRISPR-mediated editing of *Pxkmo* exon 3, of which the cutting efficiency (estimated probability of generating a frameshift mutation with a given sgRNA [26]) was predicted as 73.3 and 67.8% respectively. For the knockout of *Pxcardinal*, *cad*-sgRNA1 (expected cleavage efficiency = 62.0%) and *cad*-sgRNA2 (expected cleavage efficiency = 60.9%) were selected to target exon 6 (Fig. 2a).

As a preliminary assessment of whether *Pxkmo* and *Pxcardinal* genome sequence could be edited using the chosen sgRNAs, we first conducted in vitro cleavage assays using Cas9 protein and corresponding sgRNAs. Individually, each sgRNA targeting *Pxkmo* or *Pxcardinal* was combined with Cas9 protein in vitro and used to treat wildtype genomic DNA fragments of the target area in PCR tubes. In all cases, bands of the size expected for successful cleavage events were observed via gel electrophoresis (Fig. S 1A). Specifically, for *Pxkmo*, 120 bp and 583 bp bands were generated with *kmo*-sgRNA1; 270 bp and 433 bp bands with *kmo*-sgRNA2. For *Pxcardinal*, 662 bp and 220 bp bands were created with *cad*-sgRNA1, while 696 bp and 186 bp bands were produced with *cad*-sgRNA2. In addition, embryonic injection was conducted with sgRNAs/Cas9 mixtures, and the T7E1 assay of *Pxkmo* and *Pxcardinal* G_0_ mosaic adults showed the same pattern of bands as those resulting from the in vitro cleavage assay (Fig. S 1B). These data indicated that the designed sgRNAs could be applied for CRISPR-mediated gene editing in both *Pxkmo* and *Pxcardinal* targets.

### Germline disruption of *Pxkmo* and *Pxcardinal*

**Fig. 3:**
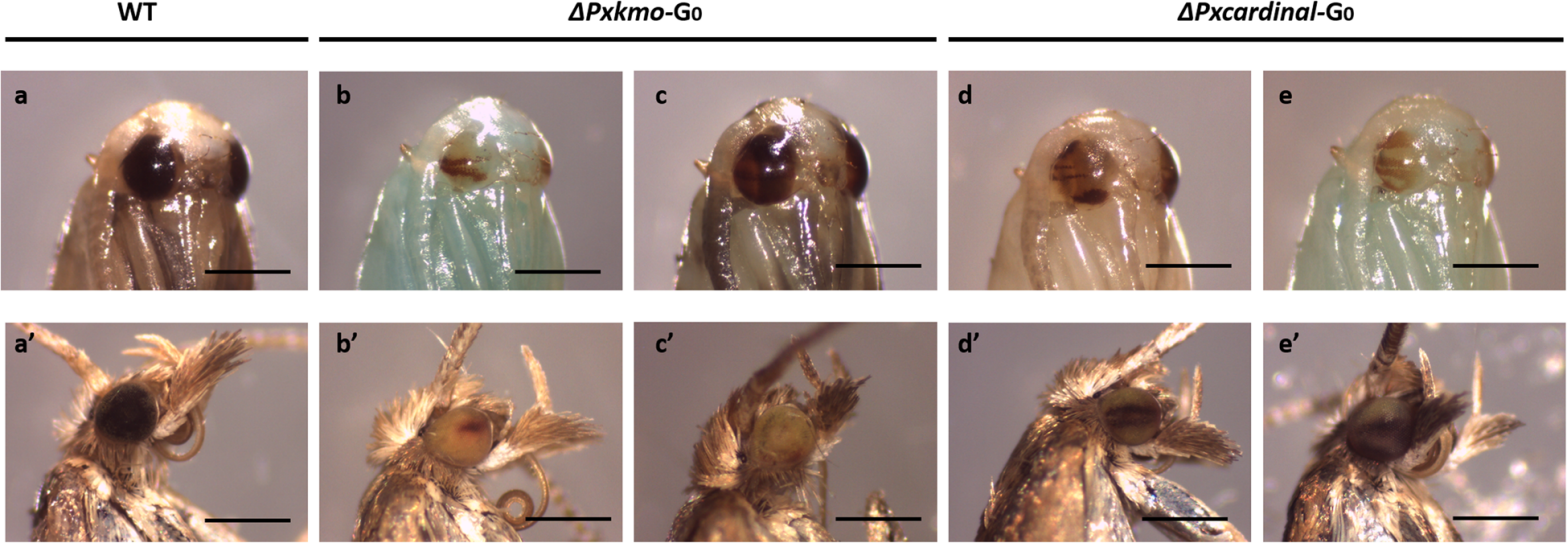
Representative phenotypes of G_0_ mosaics. Pupae (a-e) and adults (A’-E’). A and A’: wildtype (WT) control. B, B′, C and C′: *Pxkmo* mosaic mutants. D, D’, E and E’: *Pxcardinal* G_0_ mosaic mutants. Differences of pupal body color were due to differences in developmental stages, while patchy eye pigmentation was linked to gene editing. Scale bar = 0.5 mm

In total, 222 and 182 eggs were injected with *Pxkmo* and *Pxcardinal* sgRNA mixtures, respectively. For each gene, both sgRNAs were combined in the same injection mix. Normally, pupal compound eyes of wildtype DBM change from translucent in early pupae to black in late pupae and then remain black during the adult stage. However, randomly patchy coloration with dark stripes appeared in pupal and adult compound eyes of both *Pxkmo* (71.3%) and *Pxcardinal* (56.4%) G_0_ founders (Table 1 and Fig. 3). Sanger sequencing of these mosaics post egg-laying confirmed successful editing events of both genes (Fig. S 1C and D) by showing multiple peaks at the predicted cleavage sites.

**Table 1:** Mutagenesis of *Pxkmo* and *Pxcardinal* using CRISPR/Cas9 system

	G_0_			G_1_		
	Injected eggs	Surviving pupae (%)	Visible mosaic adults (%)	Total number of G_0_s crossed (pools)	G_0_ pools showing mutant G_1_	Minimal germline transformation efficiency
*Pxkmo*	222	94 (42.3)	67 (71.3)	94 (8)	8	17.0% (16/94)
*Pxcardinal*	182	55 (30.2)	31 (56.4)	28 (8)	8	57.1% (16/28)

**Fig. 4:**
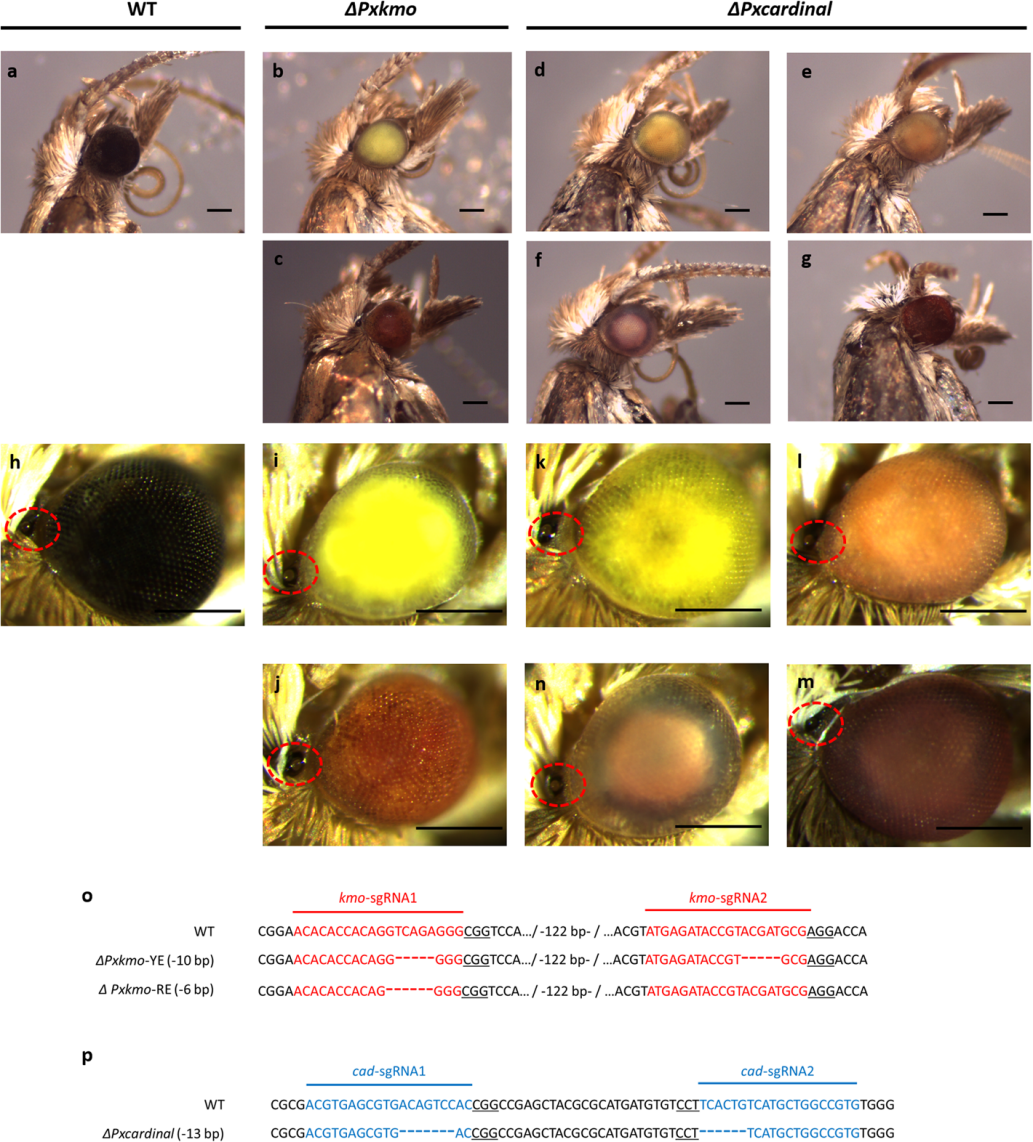
Phenotypes and genotypes of *Pxkmo* and *Pxcardinal* mutants. **a** and **h**: Wildtype phenotype. **b** and **i**: *Pxkmo* yellow-eye mutant phenotype. **c** and **j**: *Pxkmo* red-eye mutant phenotype. **d**-**g** and K-M: *Pxcardinal* mutant phenotypes. For *Pxcardinal* newly eclosed adults showed yellow compound eyes (**d** and **k**), which started to change on Day 2 or Day 3 post eclosion and gradually turned red (in the order of D to G, or K to M), although a small minority of individuals did not change. **h**-**m**: Red dashed circles indicate the positions of ocelli on increased magnification images. **o**: *Pxkmo* mutant genotypes. Two sgRNA targets are shown with red color. **p**: *Pxcardinal* mutant genotypes. Two sgRNA targets are marked with blue color. The protospacer adjacent motif (PAM) sites are underlined. Abbreviation: YE: yellow-eye; RE: red-eye. Scale bar = 0.2 mm

To validate the presence of germline mutations, 8 pools of *Pxkmo* G_0_s (94 moths in total with equal number of males and females in each pool), were set up for mating. Two strikingly different mutant phenotypes, yellow and red compound eyes were observed in the G_1_ adults derived from all 8 pools (phenotypes were consistent with the G_3_ mutants shown in Fig. 4b and c while wildtype eyes were shown in Fig. 4a and h). Under the conservative assumption that only 2 adults (a male and a female) in each G_0_ pool carried germline mutations, the minimum *Pxkmo* germline editing efficiency was estimated as 16.7% (16/96) (Table 1). In a similar procedure, 8 pools of *Pxcardinal* G_0_s were set up for crossing (28 moths in total with equal number of males and females in each pool). In contrast to *Pxkmo* injections, here only one mutant phenotype was observed in the G_1_ generation (yellow compound eye mutants consistent with G_3_ mutant phenotype in Fig. 4d) - comprising 57.1% minimal editing efficiency (Table 1).

**Fig. 5:**
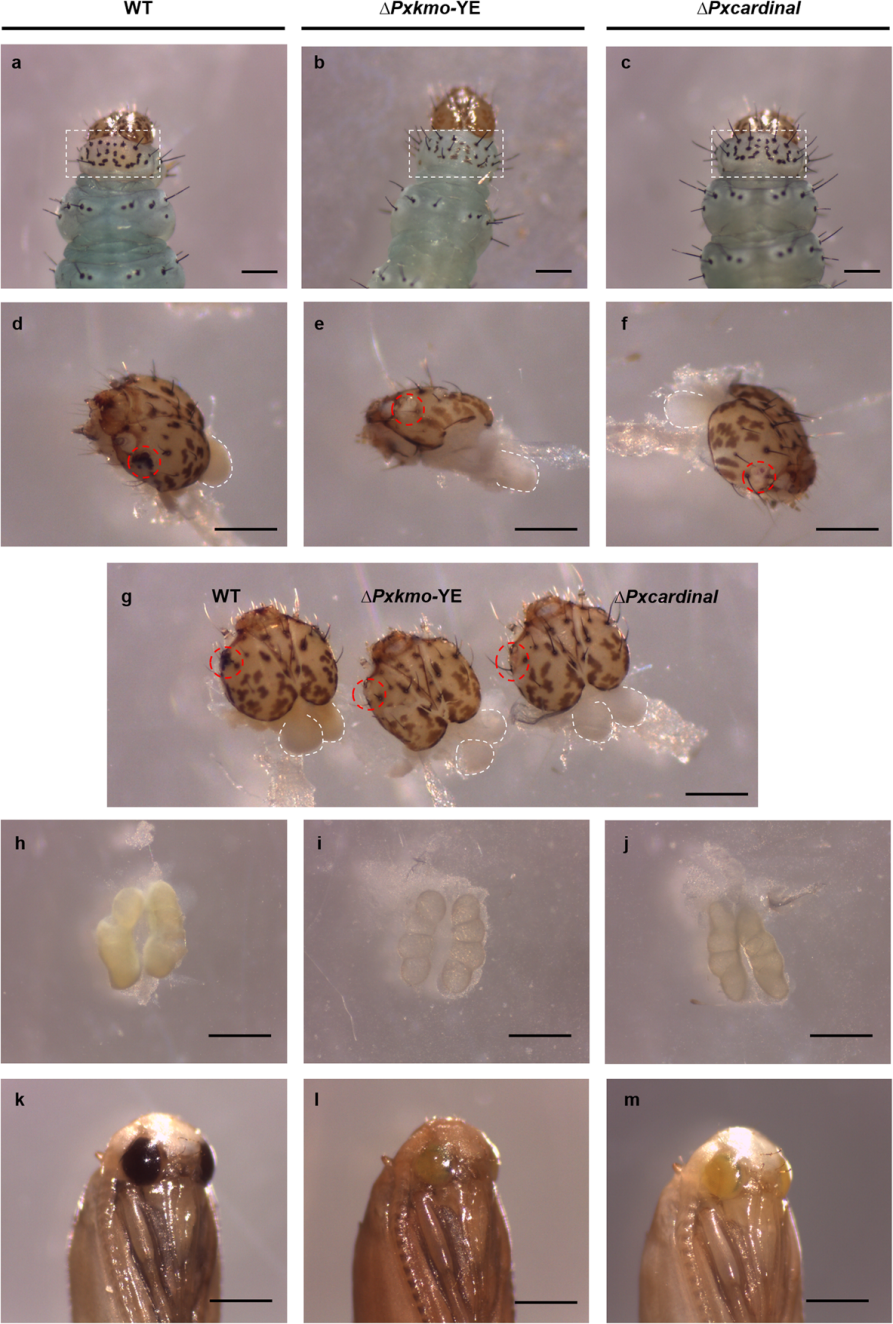
The 4th instar larvae and pupae of *Pxkmo* and *Pxcardinal* yellow-eye knock-out lines. **a**-**c**: larval body including head and partial thorax. **d**-**g**: dissected larval heads. **h**-**j**: dissected larval testes. **a**, **d** and **h**: wildtype larva. **b**, **e** and **i**: *Pxkmo* larva. **c, f** and J: *Pxcardinal* larva. White dash rectangles are used to indicate dorsal protothorax of larvae. Dissected brains are highlighted with white dashed lines while colorless ocelli are indicated with red dashed circles. **a, b, c** and **g**: dorsal view of larval heads. **d**-**f**: lateral view of larval heads. K: wildtype pupa. **l**: *Pxkmo* pupa. **m**: *Pxcardinal* pupa. Scale bar = 0.5 mm

To generate single mutant-allele homozygous knockout lines of *Pxkmo* and *Pxcardinal* for further analysis, identified G_1_ mutants of the three phenotypes (*Pxkmo* yellow or red-eye and *Pxcardinal* yellow-eye) were first outcrossed to wildtype in single-pairs producing G_2_ family pools of heterozygous black-eyed offspring. For each of these G_2_ family pools, males and females were sib-crossed in pairs. Identified eye-mutant G_3_ individuals from these sib-pair crosses were isolated and subsequently sib-crossed again in pairs to give G_4_ family pools. Once G_4_ eggs had been laid, a single G_3_ parental pair for each of the three different phenotypes was sequenced to assess whether they were homozygous with regards to their respective mutant allele. In each case, only one allele was observed in each parental pair, suggesting that the process of wildtype outcrossing and subsequent sib-crossing was successful in bringing together the same mutant alleles in these G_3_ parental pairs chosen for sequencing. For the *Pxkmo* yellow-eye parents, this single allele consisted of two 5 bp deletions (− 10 bp in total) found respectively in the *kmo*-sgRNA1 and *kmo*-sgRNA2 target sites, causing a frameshift and early translation stop signal in *Pxkmo*. In contrast, a 6 bp deletion in the *kmo*-sgRNA1 target region, causing a 2 aa in-frame deletion, was detected in the *Pxkmo* red-eye mutant parents (Fig. 4o). Sequencing results of the *Pxcardinal* yellow-eye parents also exhibited a single allele with a 7 bp deletion in the *cad*-sgRNA1 and a 6 bp deletion at the *cad-*sgRNA2 target sites (− 13 bp in total) (Fig. 4p). Offspring from these sequenced G_3_ parents were subsequently maintained as three homozygous knockout lines. It was noted that the eye color of most *Pxcardinal* yellow-eye mutant adults changed from yellow to red from the 2nd or 3rd day post eclosion, although a small minority of individuals remained yellow over time (Fig. 4d-g). Such a color shift over time was not observed in the *Pxkmo* yellow-eye strain. It was further noted that ocelli (eye-spot) color in *Pxkmo* and *Pxcardinal* mutant adults was lighter than in wildtype individuals (Fig. 4h-m) but obviously darker than their larval form, where it appeared white/colorless (Fig. 5 d-f).

After outcrossing the *Pxkmo* yellow-eye strain with wildtype, only wildtype black compound eyes could be observed in the heterozygous offspring, indicating that yellow-eye was a recessive phenotype in *Pxkmo* (Fig. S 2A). Similarly, outcrossing of *Pxkmo* red-eye mutants with their wildtype counterparts also resulted in only black eyes, which again confirmed that *Pxkmo* was a dominant gene (Fig. S 2B). However, when *Pxkmo* yellow-eye and *Pxkmo* red-eye individuals were crossed, only red-eye offspring were observed, illustrating that red-eye was dominant over yellow-eye in *Pxkmo* mutants (Fig. S 2C). Similarly, wildtype black compound eyes were also found in heterozygous *Pxcardinal* mutant after crossing the *Pxcardinal* line with wildtype, showing that the induced *Pxcardinal* mutant allele was also recessive (Fig. S 2D). We further noted that no observable difference was found between male and female adult compound eyes in these heterozygous mutants, both *Pxkmo* and *Pxcardinal* genes should not be sex-linked (i.e. located on the Z chromosome) in DBM.

### Larval and pupal phenotypes of *Pxkmo* and *Pxcardinal* yellow-eye lines

For a better understanding of mutant phenotypes caused by frame-shift disruption of *Pxkmo* and *Pxcardinal*, both yellow-eye lines were further investigated. In wildtype DBM, during late embryonic and all larval stages, a patch of yellow coloration could be observed on the back of the protothorax but was absent in both *Pxkmo* and *Pxcardinal* mutants (Fig. S 3). Representative phenotypes of 4th instar larvae are shown in Fig. 5a-c. Note that colorless ocelli were found in these two mutant lines in comparison with black ocelli in their wildtype counterparts (Fig. 5). By dissecting 4th instar larvae, we found that the pigmentation of larval brains was darker in wildtypes (dark yellow) than in *Pxkmo* and *Pxcardinal* mutants (white), while no observable difference was found between *Pxkmo* and *Pxcardinal* mutants (Fig. 5d-g). Therefore, we propose that the absence of yellow pigmentation on the mutant dorsal protothorax was caused by whitish larval brain coloration. Compared with the yellow/white testes in wildtype 4th instar larvae, the color of testes become transparent in *Pxkmo* while semi-transparent in *Pxcardinal* mutants (Fig. 5h-j). At the pupal stage, compound eyes of wildtypes usually changed from translucent to black over time. However, compound eyes of both *Pxkmo* and *Pxcardinal* mutants turned yellow instead of black in late pupae (Fig. 5k-m). Additionally, no observable difference was found between male and female mutant adults (Fig. S 4).

## Discussion

Considering the ease with which they can be visually screened, eye pigmentation genes have become useful tools for identifying germline transformation and gene editing events in several insect species. Here, we identified the *P. xylostella* homologs of *kmo* and *cardinal*, which have been reported as important genes involved in ommochrome synthesis of mosquitoes and silkworm [13, 20, 24] and characterized their mutant phenotypes using the CRISPR/Cas9 system. Relatively high rates of somatic editing (≥ 71.3% in *Pxkmo* and ≥ 56.4% in *Pxcardinal*) and germline transformation (≥ 17.0% in *Pxkmo* and ≥ 57.1% in *Pxcardinal*) were achieved, compared with our previous reports targeting different genes in *P. xylostella* [25, 27]. This was possibly due to the use of Cas9 protein rather than in vitro transcribed mRNA used previously, in each case delivered with sgRNAs into embryos. Our work validates that the CRISPR/Cas9 system is highly active in DBM for generating site-specific mutations and suggests that improvements in the system can be achieved through the use of protein components.

The ommochrome synthesis pathway in the lepidopteran model insect *B. mori*, whose eye pigmentation is solely determined by ommochromes, has been well studied [15]. There, kmo was found to be able to convert kynurenine into 3-hydroxykynurenine, which was further transferred into pigment granule by the White/Scarlet heterodimer. Kynurenine and 3-hydroxykynurenine could both function as yellow pigment. 3-hydroxykynurenine was further auto-oxidized into xanthommatin, a process that could also be catalyzed by Cardinal. The oxidized form of xanthommatin exhibited yellow coloration but turned red under reductive conditions. Additionally, Cardinal participated in the synthesis of silkworm ommin, responsible for brown pigmentation [15]. Thus, wildtype black eye coloration is a combination of all these pigments. It has been reported in *B. mori* that the disruption of *kmo* (*w-1* strain) causes a white-eye phenotype, consistent with the mutant phenotype of other ommochrome pathway genes *scarlet* (*w-2* line) and *white* (*w-3* line) [18, 19]. Similar white-eye phenotypes were also reported in *kmo*-deficient mutants of *T. castaneum* and mosquitoes [12, 20, 24]. It is noted that the above mutants in *B. mori* were also accompanied with color changes in egg shell. Although we observed a difference in ‘egg color’ between wild-type and knockout strains of DBM (Fig. S3), we identified the source of this color change in DBM as being due to differences in the somatic pigmentation of the developing embryo/neonate. Even in wild-type DBM, the egg shell is completely transparent (allowing observation of the developing embryo) and as such we did not expect - and did not observe – the knockout-associated chorion color changes which are present in *B. mori*.

Our work shows that CRISPR-induced mutagenesis of *Pxkmo* resulted in yellow or red compound eyes. Further sequencing confirmed that the yellow-eye phenotype was linked to a frame-shift disruption of the *Pxkmo* coding region, a presumed amorphic allele likely resulting in either mRNA degradation before translation or the generation of a truncated polypeptide due to the presence of a premature in-frame stop codon. It is probable that in the absence of functional Pxkmo protein, 3-hydroxykynurenine and its metabolite xanthommatin could not be synthesized, leading to the loss of yellow and red pigments at the terminal steps of the ommochrome pathway. Therefore, the ocular yellowing in *Pxkmo* knockout adults likely resulted from the accumulation of kynurenine, which might be removed from *B. mori w-1* by participating in other metabolite pathways [15, 28]. Sequence analysis also revealed that the *Pxkmo* red-eye phenotype was linked to an in-frame mutation with the absence of only two amino acids. This possibly reduced *Pxkmo* protein activity to some extent due to the change of protein structure within the FAD binding domain, but some ommochromes were still synthesized to form the red-eye phenotype, which means the red-eye mutant allele was likely hypomorphic. However, further investigation (e.g. western blot or Immunoelectrophoresis) is needed to confirm whether *Pxkmo* protein existed in yellow-eye or red-eye lines and quantify their differences. In addition, individual conserved domains of both proteins could be edited with CRISPR to further investigate their functions.

In the current work, a presumed amorphic allele of *Pxcardinal*, caused by a frameshift mutation with a total 13 bp deletion in its coding sequence, resulted in a yellow-eyed mutant which gradually shift to red as adults aged. This could be explained by the slow auto-oxidation of yellow pigment 3-hydroxykynurenine without the catalysis of *Pxcardinal* protein, and the eventual accumulation of red pigment xanthommatin. A similar change of compound eye coloration during adult stages has also been described in *Culex pipiens*, however the mutant mosquito eyes turned from red to wildtype black as adults aged [12], while our *Pxcardinal* mutants changed from yellow to red and never reverted to wildtype black. In contrast, *cardinal*-defective *B. mori* only showed red eyes at their adult stage [13]. Additionally, in both *C. pipiens* and *B. mori*, larval ocelli showed significant color change from colorless to red during their development, which was not observed in the *Pxcardinal* knockout line generated here.

Our dissections showed a lighter color of *Pxkmo* and *Pxcardinal* mutant larval brains compared with wildtype, a divergence which, to our knowledge, has not been described in other species. This phenomenon was possibly caused by the accumulation of ommochrome pathway pigments in larval brains of wildtype larvae and the lack of such pigments in *Pxkmo* and *Pxcardinal* mutants. In addition, testes of *Pxkmo* and *Pxcardinal* larvae were visibly different from wildtypes, indicating that ommochromes might also participate in testes pigmentation, consistent with previous research in *Ephestia kuehniella* [29]. It was further noted that both *Pxkmo* and *Pxcardinal* larval ocelli were colorless but darkened at adult stages, albeit always remaining lighter than wildtype individuals. This phenotype indicates that the same pigments are likely involved in the coloration of both adult ocelli and compound eyes.

Although ommochromes are the only eye pigments thought to be involved in *B. mori*, the situation in DBM remains unexplored. The presence of pteridine pathway products in *Drosophila* have been reported to exhibit yellow and red pigmentation [30]. Putative homologs of *Drosophila* pteridine synthesis pathway genes, such as *punch* and *purple*, could also be found in DBM with BlastP. Therefore, it is possible that the pteridine synthesis pathway exists in DBM and was revealed when ommochromes were absent in *Pxkmo* or *Pxcardinal*-deficient adult compound eyes, providing the yellow pigmentation observed. This would be consistent with early reports in the other lepidopteran *Ephestia kuehniella*, whose red eye mutant ‘a’ (lacking ommochrome accumulation) was caused by pteridines [29, 31, 32]. Similar evidence could also be found in *Helicoverpa armigera* [33]. Further functional investigation of other ommochrome and pteridine pathway genes is needed to assess this possibility in DBM. Complementary biochemical approaches, such as high-performance liquid chromatography–mass spectrometry (HPLC-MS), could be used to identify and quantify pigments in DBM eyes, which should further increase our understanding of eye pigmentation pathways in this lepidopteran pest.

This is the first report detailing the functional validation of eye pigmentation genes in DBM. Additionally, these *Pxkmo* and *Pxcardinal* yellow-eye/red-eye mutant strains have been readily kept in our lab for over 6 generations, indicating no severe fitness costs to individuals bearing null-allele mutations in these genes. Therefore, the *Pxkmo* and *Pxcardinal* genes investigated here can potentially be used as convenient visual markers for germline editing of DBM. In future work, *Pxkmo* and *Pxcardinal* could be targeted for site-specific knock-in of sgRNA-coding cassettes to construct in vivo sgRNA expressing strains. Combined with existing germline-expressing Cas9 transgenics [34], this would allow the further exploration of utilizing the powerful gene drive system as a pest management tool to control this global pest.

## Conclusions

In conclusion, using the CRISPR/Cas9 gene editing system, we identified and characterized orthologues of two important eye pigmentation genes *kmo* and *cardinal* in DBM, whose disruption resulted in novel and distinguishable mutant phenotypes. This work will not only provide potential targets for genetics-based pest control of this highly invasive, global pest, but also benefit researches on the evolution and divergence of ommochrome synthesis pathway genes across the Insecta.

## Methods

### Insect rearing

In the current study, all knockout moths were generated using the Vero Beach wildtype strain. Insect larvae were reared on beet armyworm artificial diet (Frontier Biosciences, USA) while adults were fed with 10% sugar solution. All the insects were maintained at 25 °C with 50% relative humidity under a 16 h light: 8 h dark cycle.

### Identification of putative homologs and construction of molecular phylogenetic trees

To identify putative *P. xylostella kmo* and *cardinal* homologs, protein sequences encoded by *B. mori kynurenine 3-monooxygenase* (accession number: ABY73874.1) and *D. melanogaster cardinal* (accession number: NP_651081.1) were used as blast queries against Liverpool DBM genome databases (http://lepbase.org/), and the hits were reciprocally blasted against *B. mori* and *D. melanogaster* genomes respectively (https://blast.ncbi.nlm.nih.gov/Blast.cgi). An online tool Protter (http://wlab.ethz.ch/protter/start/) was used to analyze transmembrane domains in putative *P. xylostella kmo* and *cardinal* homologs. Protein sequences of *kmo* and *cardinal* orthologs in other species including *B. mori*, *Spodoptera litura*, *Helicoverpa armigera*, *T. castaneum*, *Aedes aegypti*, *D. melanogaster* and *Nilaparvata lugens* were collected from NCBI databases (https://www.ncbi.nlm.nih.gov/) and utilized to construct phylogenetic trees with MEGA 5.1 software using the Maximum Likelihood (ML) method.

### Design of sgRNAs

Genomic DNA was extracted from wildtype adults using the NucleoSpin Tissue kit (Macherey-Nagel, Germany). Two pairs of primers were applied to clone gene fragments of *Pxkmo* (LA5000 and LA4974) and *Pxcardinal* (LA4985 and LA4986) using Q5 High-Fidelity DNA Polymerase (NEB, UK). The amplicons were purified with Monarch DNA Gel Extraction Kit (NEB, UK) and sequenced. Obtained sequences of both gene fragments were screened to find sgRNA targets using CHOPCHOP (https://chopchop.cbu.uib.no/) based on the N_20_-NGG rule [26]. Sequence information for primers used in this study is listed in Table S1.

### In vitro synthesis and cleavage assay of sgRNAs

The synthesis of all sgRNAs was carried out as described previously [24]. In brief, DNA templates for in vitro synthesis of sgRNAs, which targeted *Pxkmo* (i.e. kmo-sgRNA1 and kmo-sgRNA2) and *Pxcardinal* (i.e. cad-sgRNA1 and cad-sgRNA2), were respectively amplified using forward primers LA5001, LA5002, LA5005 and LA5006 (each primer containing a T7 promoter, 20 nt target site and a 16 nt sequence overlap with reverse primer) as well as a common reverse primer LA137. PCR amplicons were cleaned up with the Monarch DNA Gel Extraction Kit (NEB, UK). Afterwards, sgRNAs were in vitro transcribed from these templates using MEGAscript T7 transcription kit (Life Technologies, USA) and then purified with the MEGAclear Kit (Life Technologies, USA). Templates of each target gene were prepared from wildtype DNA samples with the same primer sets used in section 2.3, providing targets for Cas9 cleavage. Furthermore, an in vitro cleavage assay was conducted using the synthesized sgRNAs and commercially purchased SpCas9 nuclease protein (NEB, UK) following the manufacturer’s instructions.

### Embryonic microinjection

Wildtype DBM adults were kept in a 15 cm × 15 cm × 15 cm net cage for mating, and glass slides covered with cabbage juice were set inside the cage for oviposition. Embryos were collected within 30 min post oviposition, followed by the injection with a mixture containing 300 ng/μl Cas9 protein (PNA Bio, USA), 150 ng/μl of each sgRNA (making the total concentration of sgRNAs at 300 ng/μl), 1 x injection buffer [35] and made up to 20 μl using nuclease-free H_2_0. The same injection process was conducted for both *Pxkmo* and *Pxcardinal* knockout experiments. The injection mixture inside each needle was only used for 1 h post loading to avoid potential degradation. Glass slides with injected eggs were kept in Petri dishes which contained small pieces of wet cotton wool in order to maintain humidity. Cotton wool was removed after 48 h post injection and artificial diet was then put in the Petri dishes to feed hatched larvae.

### Germline gene editing screening

The pupae and adults of injected G_0_s were screened for abnormal eye pigmentation. Genomic DNA of G_0_ mosaic adults was extracted after they had laid eggs in order to amplify target regions (the same primer sets as described in 2.4); target region PCR products were gel-purified and sequenced. In addition, a T7E1 assay was conducted on these PCR products using T7 endonuclease (NEB, UK) based on manufacturer’s instructions to confirm CRISPR/Cas9 induced mutations.

For each gene editing experiment, G_0_ adult injection survivors were crossed *en masse* in multiple pools, and any identified G_1_ mutant adults were subsequently pair crossed to wildtype. The heterozygous G_2_ individuals derived from the same parents were sib-crossed to generate G_3_s; offspring with non-wildtype eye color were collected and maintained as mutant lines. Dissection of 4th instar larvae of these strains was conducted to assess color changes to the brains and testes. Most phenotypic images were taken with a Leica E24 HD stereo microscope (Leica Microsystems, Germany), except for photos of adult ocelli which were taken using a Leica MZ165FC microscope (Leica Microsystems, Germany).

## Supplementary information

**Additional file 1: Table S1.** Primers used in current study. **Fig. S1.** CRISPR-mediated gene editing in G_0_ founders. **Fig. S2.** Phenotypes of *Pxkmo* and *Pxcardinal* heterozygous mutants derived from different crosses. **Fig. S3.** Phenotype of *Pxkmo* and *Pxcardinal* yellow-eye knock-out lines. **Fig. S4.** Phenotypes of *Pxkmo* and *Pxcardinal* G_1_ mutations.

## Data Availability

The datasets and materials used and/or analyzed during the current study are available from the corresponding authors or reasonable request.

## Abbreviations

DBM: Diamondback moth.
*kmo*: *Kynurenine 3-hydroxylase*
