# Supplementary material for: Ommochrome pathway genes kynurenine 3-hydroxylase and cardinal participate in eye pigmentation in Plutella xylostella
Source: BMC Mol Cell Biol. 2020 Sep 11;21:63. doi: 10.1186/s12860-020-00308-8 (PMC7488671; doi:10.1186/s12860-020-00308-8)

# **Supplementary files**

**Table S 1 Primers used in current study**.

Figure S 1 CRISPR-mediated gene editing in G_0_ founders. A: *in vitro* cleavage with synthesized sgRNAs. Wildtype target amplicons were treated with each sgRNA, while non-treated wildtype amplicons were used as negative control (named Wildtype). B: T7E1 assay of G_0_ injected embryo pools. M: NEB 1kb Plus DNA ladder. ‘+’ and ‘-’ indicate with and without T7 endonuclease treatment. Expected band sizes derived from correct cleavage in target sites are marked with red (*Pxkmo*) and blue (*Pxcardinal*) triangles. C and D: sequencing results of G_0_ pools. Sequencing orientation is indicated at the right of the figure. Multiple peaks in G_0_ sequencing result illustrate the presence of different genotypes which were most likely caused by CRISPR editing.

Figure S 2 Phenotypes of *Pxkmo* and *Pxcardinal* heterozygous mutants derived from different crosses. A: *∆Pxkmo-YE* x WT; B: *∆Pxkmo-RE* x WT; C: *∆Pxkmo-YE* x *∆Pxkmo-RE*; D: *∆Pxcardinal* x WT. Abbreviation: WT: wildtype control; *∆Pxkmo-YE*: *Pxkmo* yellow-eye mutant; *∆Pxkmo-RE*: *Pxkmo* red-eye mutant; *∆Pxcardinal*: *Pxcardinal* knockout mutant. Scale bar: 0.2 mm.

Figure S 3 Phenotype of *Pxkmo* and *Pxcardinal* yellow-eye knock-out lines. The yellow pigmentation which existed in wildtype but not in mutant lines is indicated with yellow triangles.

Figure S 4 Phenotypes of *Pxkmo* and *Pxcardinal* G_1_ mutations. Male and female individuals are indicated with ♂/♀symbols. Abbreviation: WT: wildtype control; *∆Pxkmo*: *Pxkmo* knockout mutants. *∆Pxcardinal*: *Pxcardinal* knockout mutants. Scale bar: 0.5 mm.

| Primer names | Primer sequences (5’-3’) | Amplification products |
| --- | --- | --- |
| LA5000 | TTGTATCTAACGTCCTTCGCCTCC | Gene fragment flanking *Pxkmo* targets |
| LA4974 | ATTCCGCCCTACGGAGTAGATG |  |
| LA4985 | AGGACAGACCTGTCTAACCGC | Gene fragment flanking *Pxcardinal* targets |
| LA4986 | AGCTATGTGGCTGGATGTCGG |  |
| LA5001 | gaaattaatacgactcactataggACACACCACAGGTCAGAGGGgttttagagctagaaa | *kmo*-sgRNA1 template |
| LA5002 | gaaattaatacgactcactataggATGAGATACCGTACGATGCGgttttagagctagaaa | *kmo*-sgRNA2 template |
| LA5005 | gaaattaatacgactcactataggACGTGAGCGTGACAGTCCACgttttagagctagaaa | *cad*-sgRNA1 template |
| LA5006 | gaaattaatacgactcactataggCACGGCCAGCATGACAGTGAgttttagagctagaaa | *cad*-sgRNA2 template |
| LA137 | AAAAGCACCGACTCGGTGCCACTTTTTCAAGTTGATAACGGACTAGCCTTATTTTAACTTGCTATTTCTAGCTCTAAAAC | Common reverse primer for sgRNA synthesis |


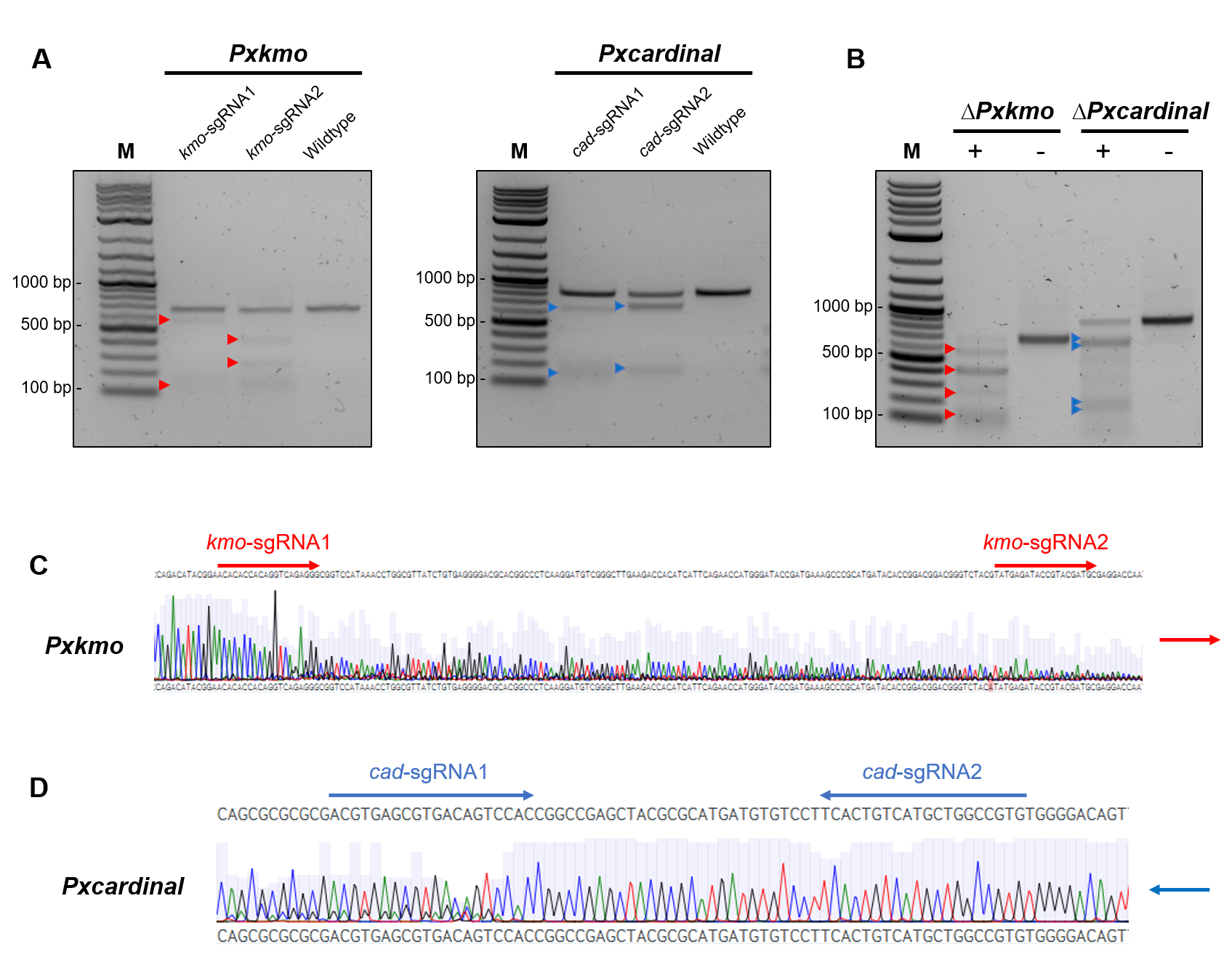


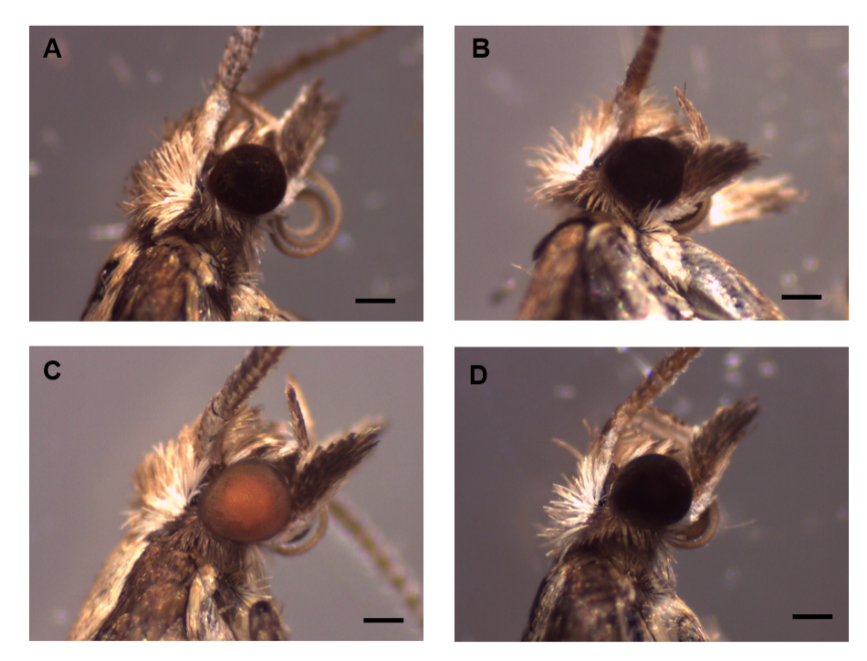


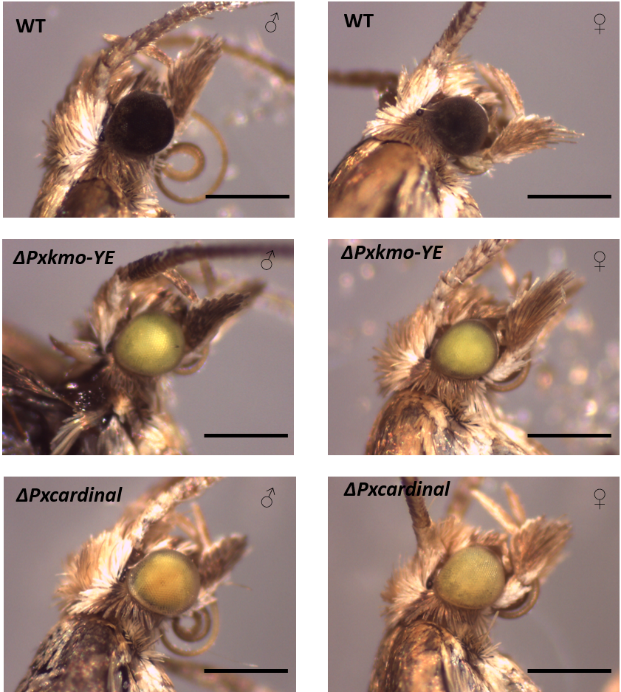

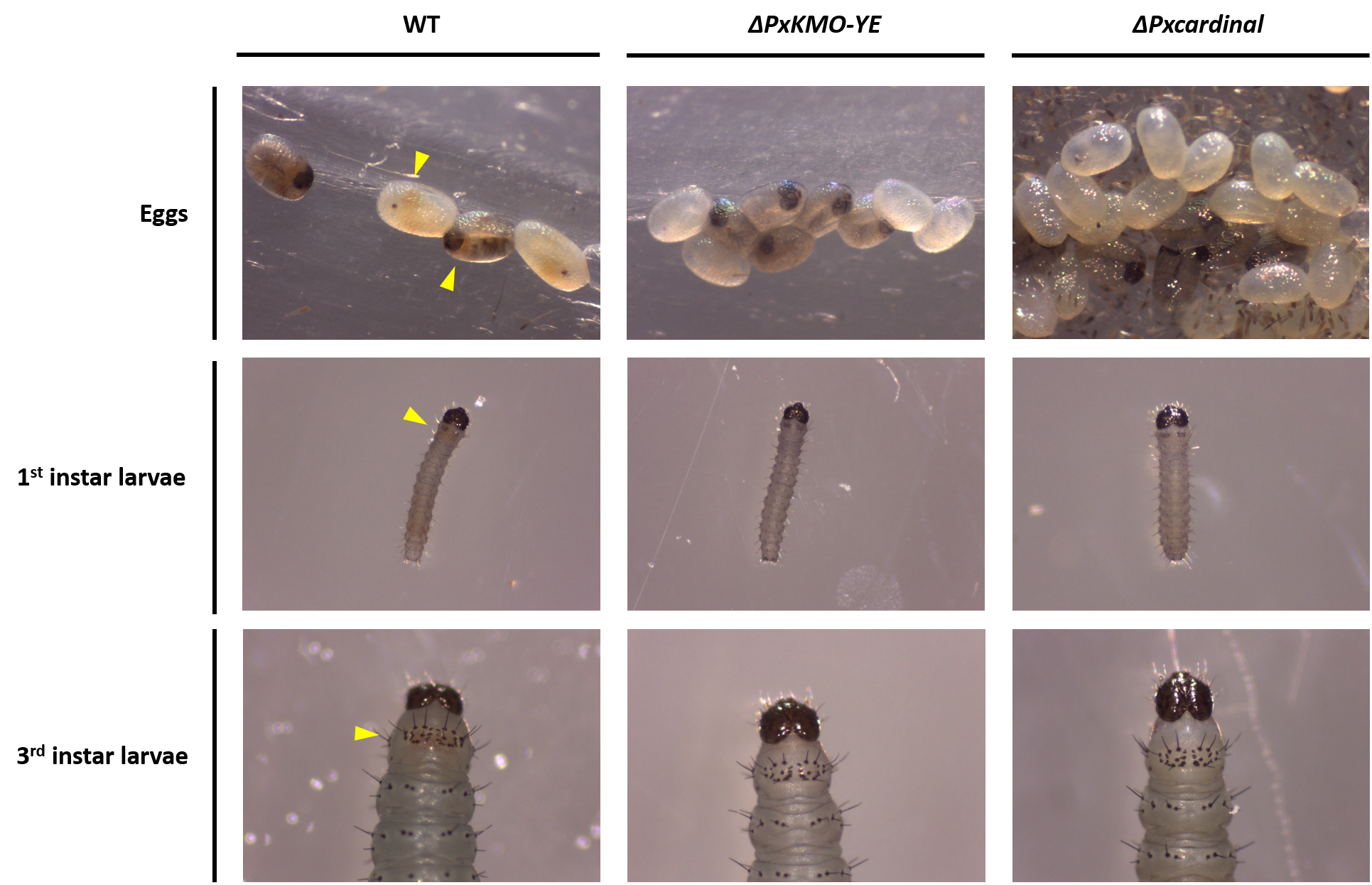

Supplement: Supplementary file 1 — Additional file 1: Table S1. Primers used in current study. Fig. S1. CRISPR-mediated gene editing in G0 founders. Fig. S2. Phenotypes of Pxkmo and Pxcardinal heterozygous mutants derived from different crosses. Fig. S3. Phenotype of Pxkmo and Pxcardinal yellow-eye knock-out lines. Fig. S4. Phenotypes of Pxkmo and Pxcardinal G1 mutations. [file 12860_2020_308_MOESM1_ESM.docx]
